# Silencing *PPP2R1A* inhibits the progression of gastric cancer cells

**DOI:** 10.1007/s00432-025-06177-y

**Published:** 2025-04-18

**Authors:** Gengming Cheng, Laibijiang Wusiman, Dingding Song, Wenbin Zhang

**Affiliations:** Gastrointestinal Surgery Department, Xinjiang Medical University Affiliated Cancer Hospital, Xinjiang Uygur Autonomous Region, Urumqi, 830011 People’s Republic of China

**Keywords:** *PPP2R1A*, Gastric cancer, Cell proliferation, Cell migration and invasion, Cell apoptosis

## Abstract

**Background:**

Protein phosphatase 2 regulatory subunit A alpha (*PPP2R1A*) is the most common scaffold protein in the PP2A complex and has known tumor-suppressive functions. However, its role in gastric cancer (GC) is still unclear. This study aims to elucidate the potential regulatory role of *PPP2R1A* in the biological functions of GC.

**Methods:**

The mutation status and expression levels of *PPP2R1A* in GC were assessed through bioinformatics analysis, the correlation between *PPP2R1A* levels and patient survival rates was examined, and its potential functional network was analyzed. Stable AGS and MGC803 cell lines were set up for overexpressing and silencing *PPP2R1A*. The effects on cell proliferation, migration, invasion, and apoptosis were assessed through CCK-8 assays, scratch assays, Transwell assays, and flow cytometry.

**Results:**

The expression of *PPP2R1A* is significantly elevated in GC samples (*P* < 0.001) and is not caused by mutations in *PPP2R1A* (*P* > 0.05). Patients with high levels of *PPP2R1A* have a poorer 5-year survival rate (*P* < 0.001). Silencing *PPP2R1A* significantly inhibits the proliferation, migration, and invasion of GC cells while promoting apoptosis (*P* < 0.01). In contrast, overexpression of *PPP2R1A* does not have a significant impact on these cellular functions (*P* > 0.05).

**Conclusion:**

*PPP2R1A* has potential oncogenic properties in the progression of GC, and knocking down the expression of *PPP2R1A* can inhibit the tumor progression of GC cells. This suggests that *PPP2R1A* may serve as a potential prognostic marker and therapeutic target for GC.

**Supplementary Information:**

The online version contains supplementary material available at 10.1007/s00432-025-06177-y.

## Introduction

Gastric cancer (GC), particularly prevalent in East Asia, is one of the most common and lethal cancers globally (López et al. 2023). In 2022, GLOBOCAN reported over 968,000 new cases and 660,000 deaths from GC, ranking it fifth in both incidence and mortality (Bray et al. 2024). The high incidence and mortality rates of GC place a significant strain on healthcare resources and societal well-being (Siegel et al. 2025). The subtle early symptoms of GC often lead to a late diagnosis. Traditional treatments are frequently limited in effectiveness and associated with significant side effects, making it difficult to improve the prognosis for these patients, with a five-year survival rate of less than 20% (Ilic and Ilic 2022). In recent years, our understanding of the molecular mechanisms underlying GC has deepened. It has been demonstrated that the dysregulation of cellular signaling pathways is closely associated with the development and progression of GC (Matsuoka and Yashiro 2024). These research advancements have highlighted the potential of immunotherapy and molecularly targeted therapies as promising new directions in the treatment of GC (Deshmukh et al. 2025; Yang et al. 2024).

Protein Phosphatase 2A (PP2A) is a major serine/threonine phosphatase that regulates diverse cellular signaling pathways through dephosphorylation, playing a critical role in tumor initiation, progression, prognosis, and treatment (Neale et al. 2025). Protein phosphatase 2 regulatory subunit A alpha (*PPP2R1A*) functions as a key scaffold protein in the PP2A complex, binding to catalytic and regulatory subunits to modulate the activity and specificity of the holoenzyme (Peris et al. 2023; Goguet-Rubio et al. 2020). Otto Kauko *et al.* demonstrated that knocking down *PPP2R1A* expression specifically impacts threonine dephosphorylation, thereby inhibiting PP2A activity. This highlights its distinct and non-redundant role compared to other PP2A inhibitory proteins such as CIP2A, SET, and PME-1 (Kauko et al. 2020). Additionally, the phosphorylation of Tyr261 in *PPP2R1A* enhances the activity of PP2A, leading to the dephosphorylation of IMPDH2, thereby promoting S-phase progression and tumor growth downstream of the FGFR signaling pathway (Zhou et al. 2025). *PPP2R1A* also mediates the dephosphorylation of hnRNPA1 and promotes TERRA accumulation, thereby facilitating telomere protection and exerting an essential oncogenic role in tumorigenesis (Sui et al. 2022). Despite extensive evidence linking *PPP2R1A* to various cancers, its specific role in GC remains to be elucidated.

In this study, we employed bioinformatics analysis and in vitro cellular experiments to elucidate the expression patterns, clinical significance, and potential biological roles of *PPP2R1A* in GC. Our findings provide novel insights into the mechanisms underlying GC tumorigenesis and underscore the critical role of *PPP2R1A* in this disease. These results lay the foundation for further exploration of *PPP2R1A* as a potential therapeutic target.

## Methods

### Bioinformatics analysis

The TIMER online tool (http://timer.cistrome.org/) was utilized to analyze the expression levels of *PPP2R1A* across various tumor types and its correlation with other genes. The Cancer Genome Atlas (TCGA) database (https://portal.gdc.cancer.gov/) was employed to download RNA sequencing (RNA-seq) data from 448 stomach adenocarcinoma (STAD) samples and mutation annotation format (MAF) data from 397 STAD samples. The Gene Expression Omnibus (GEO) database (https://www.ncbi.nlm.nih.gov/geo/) was used to obtain the gene expression matrix from the GSE54129 dataset. R (version 4.4.1) was applied to analyze the mutation status, differential expression, and gene co-expression of *PPP2R1A*, as well as to conduct Gene Ontology (GO) and Kyoto Encyclopedia of Genes and Genomes (KEGG) enrichment analyses. The STRING database (https://cn.string-db.org/) was used to construct the protein-protein interaction (PPI) network related to *PPP2R1A*, while Cytoscape (version 3.10.1) was employed for the visualization of the PPI network. The Kaplan-Meier Plotter tool (http://kmplot.com/analysis/) was utilized to investigate the association between *PPP2R1A* expression levels and the 5-year overall survival (OS), progression-free survival (PFS), and post-progression survival (PPS) of GC patients.

### Plasmid construction

Cloning primers specific to the full-length coding sequence (CDS) of human *PPP2R1A* (Gene ID: 5518) were designed via Oligo 7 (version 7.60) and synthesized by Sangon Biotech (Shanghai, China). Total RNA was isolated from cells via TRIzol reagent (Invitrogen, #15596026, USA), and reverse-transcribed into cDNA via a reverse transcription kit (Takara, #6210A, Japan). The PCR reaction mixture included 1 µL PrimeSTAR GXL DNA Polymerase (Takara, #R050A, Japan), 10 µL 5× PrimeSTAR GXL Buffer, 4 µL dNTP Mixture, 1 µL PPP2R1A-specific cloning primers, and 500 ng cDNA template, with distilled water added to a final volume of 50 µL. The reaction was carried out in a PCR thermocycler (Bio-Rad, T100^TM^, USA) under the following conditions: an initial denaturation at 98°C for 5 min, followed by 35 cycles of denaturation at 98°C for 10 sec, annealing at 60°C for 15 seconds, and extension at 68°C for 2 min, with a final extension at 68°C for 5 min. the products were analyzed by electrophoresis and purified via FastPure DNA Mini Columns-G (Vazyme, DC301, China). The purified PCR products and pLEX-MCS plasmid (Thermo Fisher, #OHS4735, USA) were digested with *Xho I* (NEB, R0146S, USA) and *Age I* (NEB, R3552S, USA) restriction enzymes and ligated via T4 DNA Ligase (Takara, #M0202S, Japan). The ligation mixture was transformed into *E. coli* DH5α (Solarbio, #C1100, China), and positive clones were selected and sequenced using a plasmid extraction kit (QIAGEN, #12143, Germany). The PPP2R1A-specific cloning primers are as follows: pLEX-PPP2R1A-Forward: 5′-CCGCTCGAGGAAAGGGACGGAGCCAAGATG-3′; pLEX-PPP2R1A-Reverse: 5′-GACCGGTTCATACCCATACGACGTCCCAGACTACGCTGGCGAGAGACAGAACAGT-3′.

shRNA sequences targeting the *PPP2R1A* gene were designed and subcloned into the pLVX lentiviral vector by Sangon Biotech. The sequences of the shRNA are as follows: pLVX-PPP2R1A-Sense: 5′-TACCAGGATGTGGACGTCAAATTTCAAGAGAATTTGACGTCCACATCCTGGTTTTTTTC-3′; pLVX-PPP2R1A-Antisense: 5′-TCGAGAAAAAAACCAGGATGTGGACGTCAAATTCTCTTGAAATTTGACGTCCACATCCTGGTA-3′.

### Cell culture and cell transfection

Human gastric mucosal epithelial cells (GES-1), gastric adenocarcinoma cell lines (AGS and MGC-803), and embryonic kidney cells (HEK293T) were obtained from the American Type Culture Collection (ATCC). These cells were maintained in high-glucose Dulbecco’s modified Eagle’s medium (DMEM, Gibco, #C11995500BT, USA) supplemented with 10% fetal bovine serum (FBS, Gibco, #10091148, USA) and 1% penicillin-streptomycin (Cytiva, #SV30010, USA). The cells were incubated in a CO_2_ incubator (Memmert, INCO_2_/153, Germany) at 37 °C with 5% CO_2_.

HEK293T cells were plated in 24-well plates at a density of 1×10^5^ cells per well in medium without antibiotics. The cells were incubated at 37 °C in a 5% CO_2_ atmosphere until they reached approximately 80% confluence. For the transfection process, Lipofectamine 3000 reagent (Thermo Fisher, #L3000015, USA) was utilized, incorporating 8 µg of the transfection reagent along with 2 µg of either the pLEX-MCS-*PPP2R1A* plasmid or a *PPP2R1A* shRNA plasmid per well, with an empty vector included as a negative control. After 48 h, the culture supernatant was collected, and the viral particles were harvested via centrifugation (Eppendorf, 5417R, Germany) to remove cell debris.

The viral particles were utilized to infect AGS and MGC803 cells at a multiplicity of infection (MOI) of 10. After a 24-h incubation period, the medium was replaced to eliminate any uninfected virus, and the cells were cultured for an additional 48 h. Puromycin (Solarbio, #P8230-25mg, China) was subsequently used to select the transfected cell lines, and the selection process lasted for 7 days. After the selection process, surviving cells were isolated and reseeded, and RNA and protein were extracted for subsequent functional assays.

### RT-qPCR

Total RNA was isolated and reverse-transcribed into cDNA as described in the Plasmid construction section. The qPCR setup consisted of 10 µL of 2× SYBR Green PCR Master Mix (Takara, RR820A, Japan), 1 µL of specific primers targeting *PPP2R1A* or *GAPDH*, 2 µL of the cDNA template, and water to a final volume of 20 µL. The reaction was carried out in a real-time fluorescence PCR instrument (Bio-Rad, CFX96 Touch^TM^, USA) under the following conditions: an initial denaturation at 95°C for 2 min, followed by 40 cycles of denaturation at 98°C for 15 seconds, annealing at 60°C for 20 sec, and extension at 72°C for 20 seconds. The relative expression of *PPP2R1A* was quantified via the 2^-ΔΔCt^ method. The primers used for qPCR were synthesized by Sangon Biotech as follows: PPP2R1A-qPCR-Forward: 5′-CAAAGACAACACCATCGAGCA-3′; PPP2R1A-qPCR-Reverse: 5′-GGATGCCAATCACCTCGTTC-3′. GAPDH-qPCR-Forward: 5′-GAAGGTGAAGGTCGGAGTC-3′; GAPDH-qPCR-Reverse: 5′-GAAGATGGTGATGGGATTTC-3′.

### Western blot

The cells were lysed using RIPA lysis buffer (Solarbio, #R0010, China) and PMSF (Solarbio, #P8340, China). A total of 30 µg of protein sample was mixed with loading buffer, heated, and loaded onto SDS-PAGE gels (Real-Times, #RTD6116, China) for electrophoresis. Following electrophoresis, the proteins were transferred to PVDF membranes (Labselect, #TM-PVDF-R-45, China), which were subsequently blocked overnight at 4 °C with 5% nonfat dry milk (Solarbio, D8340, China). The membranes were incubated at room temperature for 2 h with primary antibodies against PPP2R1A (Proteintech, #15882-1-AP, USA; 1:1000 dilution). After the membranes were washed, HRP-conjugated anti-rabbit secondary antibodies (Affinity, #S0001, USA; 1:5000 dilution) were added, and the samples were incubated at room temperature for 1 h. The membranes were treated with ultra-sensitive ECL detection reagents (Biosharp, #BL523B, China), and signals were captured using a gel imaging system (ProteinSimple, AlphaImager HP, USA). The protein bands were quantified using ImageJ (version 1.54f), and the gray values were normalized to those of β-actin (Affinity, # AF7018, USA; 1:3000 dilution) to calculate the relative expression levels of the PPP2R1A protein.

### CCK-8 assay

Transfected cells were plated at a density of 3000 cells per well in 96-well plates and incubated for 6 h at 37 °C in a 5% CO₂ atmosphere. At the designated time points of 0, 24, 48, and 72 h, each well received 10 µL of CCK-8 reagent (Solarbio, #CA1210-500, China) and was allowed to incubate for 2 h. The optical density (OD) at 450 nm was subsequently measured via a microplate reader (Beckman Coulter, DTX 880, USA), and the readings for each time point were recorded. Each group in the experiment was performed in quintuplicate.

### Scratch assay

Transfected cells were plated at a density of 5×10^5^ cells per well in 6-well plates and incubated at 37 °C in a 5% CO_2_ atmosphere until they reached approximately 80% confluence. Using sterile pipette tips, uniform scratches were made in the center of each well, and the medium was replaced with serum-free medium for continued incubation. The scratch area was recorded at 0, 24, and 48 h using an inverted microscope (Olympus, CKX53, Japan) and a microscope imaging system (Olympus, DP27, Japan) to capture images of the scratch. The cell migration rate was calculated using the following formula: Migration rate = (Initial scratch area - Scratch area at time point) / Initial scratch area × 100%. Each experimental group included four replicates.

### Transwell assay

Transfected cells were resuspended in serum-free medium and introduced into the upper chamber of Transwell inserts (Corning, #3422, USA; 4×10^4^ cells/well). After a 24-h incubation at 37 °C in a 5% CO_2_ atmosphere, medium supplemented with 20% serum was added to the lower chamber, and the incubation was extended for another 48 h. The cells in the lower chamber were subsequently fixed with 4% paraformaldehyde (Solarbio, #P1110, China) for 20 min, followed by staining with 0.25% crystal violet (Biosharp, #BL802A, China) for 20 min. The number of cells that had migrated and invaded was quantified via microscopy. Each experimental group consisted of five replicates.

### Flow cytometry

Transfected cells were harvested into a centrifuge tube and subjected to centrifugation at 1000 rpm for 5 min. The samples were then washed with 3 mL of PBS (Biosharp, #BL302A, China) and centrifuged again at the same speed, completing a total of three washes. The cells were subsequently stained with Annexin V-FITC (eBioscience, #11–4317-87, USA) and PE (eBioscience, #14–4120-81, USA), and the percentage of apoptotic cells was subsequently assessed via flow cytometry (BD Biosciences, BD FACSCalibur, USA).

### Statistical analysis

SPSS (version 26.0) was used for the statistical analysis in this study. For continuous variables, the Shapiro-Wilk test was employed to assess normality. For those that were normally distributed, independent samples t-tests or one-way analysis of variance (ANOVA) were conducted, with results presented as mean ± standard deviation (*x̄ ± s*). For those that were not normally distributed, the Kruskal-Wallis test was used, and results were presented as median and interquartile range (IQR). For categorical variables, the chi-square test was applied, with results presented as frequencies and percentages (%). Kaplan-Meier analysis was used to plot survival curves, and the log-rank test was performed for comparative analysis. All statistical tests were two-tailed, with *P* < 0.05 considered statistically significant.

## Results

### *PPP2R1A* expression and mutation landscape in gastric cancer

**Fig. 1:**
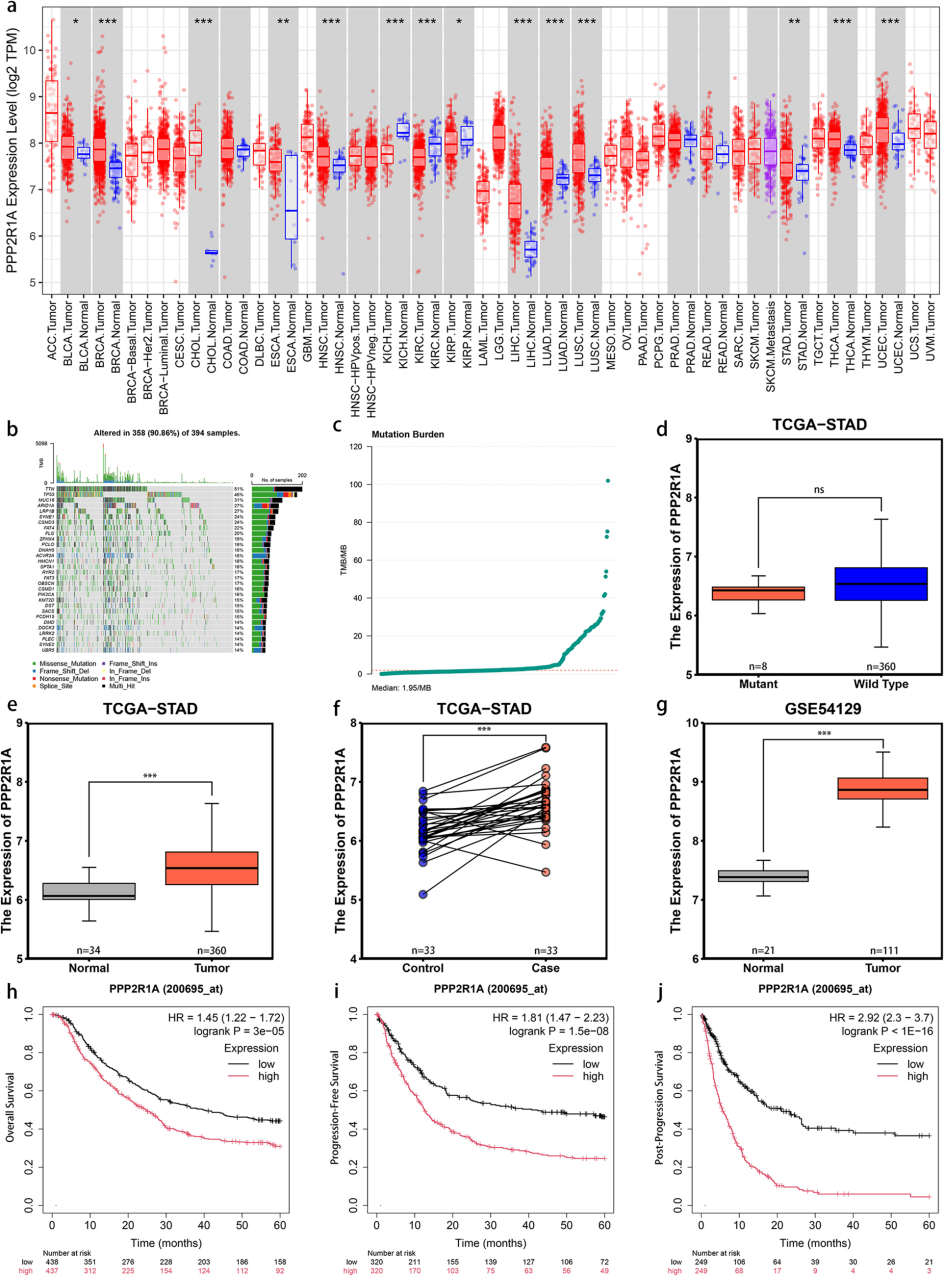
*PPP2R1A* is overexpressed in GC and is associated with poor patient prognosis. **a** Analysis of *PPP2R1A* mRNA expression levels in tumor tissues. **b** Assessment of gene mutation status in STAD patients: *n* = 394 samples. **c** Assessment of TMB in STAD patients: *n* = 394 samples. **d** Analysis of differential *PPP2R1A* mRNA expression levels between wild-type and mutated *PPP2R1A* in STAD patients: *n* = 368 samples. **e** Analysis of differential *PPP2R1A* mRNA expression levels between tumor and normal groups in wild-type *PPP2R1A* samples: *n* = 394 samples. **f** Analysis of the differential expression levels of *PPP2R1A* between case-control samples: *n* = 66 samples. **g** Analysis of differential *PPP2R1A* mRNA expression levels between tumor and normal groups in the GSE54129 dataset: *n* = 132 samples. **h**–**j** Analysis of the survival differences in five-year OS, PFS, and PPS based on high and low *PPP2R1A* mRNA expression levels in GC patients. Asterisks indicate statistically significant differences (*P < 0.05; **P < 0.01; ***P < 0.001). HR: hazard ratio

Analysis using the TIMER online tool revealed that *PPP2R1A* expression is significantly elevated in various tumor samples, including STAD (Fig. 1a; Table 1).

**Table 1:** The expression pattern of *PPP2R1A* in different tumor tissues

Tumor abbreviation	Full tumor name	PPP2R1A expression pattern	*P* value
BLCA	Bladder urothelial carcinoma	Upregulated	0.048
BRCA	Breast invasive carcinoma	Upregulated	*P* < 0.001
CHOL	Cholangiocarcinoma	Upregulated	*P* < 0.001
COAD	Colon adenocarcinoma	No significant change	0.311
ESCA	Esophageal carcinoma	Upregulated	0.008
HNSC	Head and neck squamous cell carcinoma	Upregulated	*P* < 0.001
KICH	Kidney chromophobe	Downregulated	*P* < 0.001
KIRC	Kidney renal clear cell carcinoma	Downregulated	*P* < 0.001
KIRP	Kidney renal papillary cell carcinoma	Downregulated	0.016
LIHC	Liver hepatocellular carcinoma	Upregulated	*P* < 0.001
LUAD	Lung adenocarcinoma	Upregulated	*P* < 0.001
LUSC	Lung squamous cell carcinoma	Upregulated	*P* < 0.001
PRAD	Pancreatic adenocarcinoma	No significant change	0.788
READ	Rectum adenocarcinoma	No significant change	0.246
SKCM	Skin cutaneous melanoma	No significant change	0.709
STAD	Stomach adenocarcinoma	Upregulated	0.008
THCA	Thyroid carcinoma	Upregulated	*P* < 0.001
UCEC	Uterine corpus endometrial carcinoma	Upregulated	*P* < 0.001

A total of 397 MAF samples from the TCGA database were analyzed, with mutation information extracted from 394 samples. The results showed that mutations were present in 358 samples (90.86%), with the most frequently mutated genes being *TTN* (51%), *TP53* (46%), and *MUC16* (31%) (Fig. 1b). In contrast, the mutation probability of *PPP2R1A* was only 2%. Further analysis of the tumor mutation burden (TMB) in these 394 samples revealed a median TMB of 1.95 mutations per megabase (Mb) (Fig. 1c).

Among the 368 STAD samples with both mutation and gene expression information, no significant difference was observed in *PPP2R1A* expression levels between mutated and wild-type samples (*P* > 0.05) (Fig. 1d). However, in wild-type samples, *PPP2R1A* expression was significantly higher in tumor tissues compared to normal tissues (*P* < 0.001) (Fig. 1e). Additionally, analysis of *PPP2R1A* expression data from 33 case-control samples and the dataset GSE54129 further supported that *PPP2R1A* expression is significantly upregulated in GC (*P* < 0.001) (Fig. 1f, g).

Analysis using the Kaplan-Meier Plotter tool demonstrated that GC patients with high *PPP2R1A* expression had significantly lower 5-year OS, PFS, and PPS rates compared to those with low *PPP2R1A* expression (*P* < 0.001) (Fig. 1h–j).

### *PPP2R1A* co-expression and functional networks in gastric cancer

**Fig. 2:**
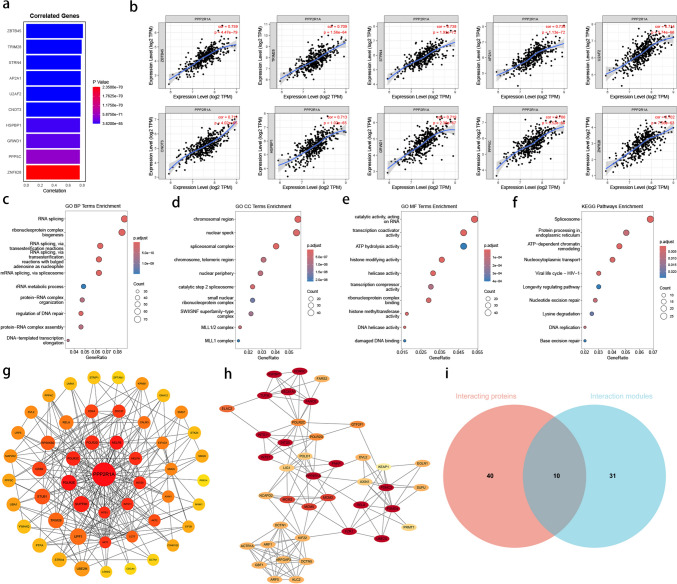
*PPP2R1A* is co-expressed with various genes and is involved in a complex functional network. **a** Evaluation of *PPP2R1A* co-expressed genes in wild-type *PPP2R1A* samples from STAD patients and list the top ten genes: *n* = 360 samples. **b** Display of the scatter plot showing the distribution of the top ten co-expressed genes with *PPP2R1A*. **c**–**f** Perform GO and KEGG enrichment analysis on the co-expressed genes of *PPP2R1A* and list the top ten entries: *n* = 899 genes. **g**, **h** Perform PPI analysis on the co-expressed genes of PPP2R1A and display the proteins that directly interact with PPP2R1A and modules in which PPP2R1A is involved in the interactions: *n* = 899 genes. **i** Display the redundancy of the interacting proteins and interaction modules of PPP2R1A. Asterisks indicate statistically significant differences (*P < 0.05; **P < 0.01; ***P < 0.001)

Gene co-expression analysis of 360 wild-type STAD samples from TCGA database revealed that *PPP2R1A* is significantly co-expressed with 898 genes (|*r*| > 0.5 and *P* < 0.05), including *ZBTB45*, *TRIM28*, *STRN4*, *AP2A1*, *U2AF2*, *CNOT3*, *HSPBP1*, *GRWD1*, *PPP5C*, and *ZNF628* (Fig. 2a). Co-expression scatter plots for *PPP2R1A* and these ten genes were generated using the TIMER online tool (Fig. 2b).

Functional enrichment analysis using Gene Ontology (GO) showed that these genes are enriched in 225 biological process (BP) terms, with top terms including RNA splicing, ribonucleoprotein complex biogenesis, and RNA splicing via transesterification reactions (Fig. 2c). In cellular component (CC) analysis, the genes were enriched in 107 terms, with chromosomal region, nuclear speck, and spliceosomal complex being the most prominent (Fig. 2d). For molecular function (MF), the genes were enriched in 77 terms, with catalytic activity acting on RNA, transcription coactivator activity, and ATP hydrolysis activity being the most significant (Fig. 2e). KEGG pathway enrichment analysis identified 13 pathways, with spliceosome, protein processing in the endoplasmic reticulum, and ATP-dependent chromatin remodeling being the top pathways (Fig. 2f).

A PPI network was constructed based on these genes, revealing that PPP2R1A directly interacts with 49 genes. MCC scoring analysis identified nine hub genes: POLR2E, NELFB, SUPT5H, POLR2C, POLR2D, INTS1, NELFA, INTS5, and INTS11 (Fig. 2g). A PPI module containing 41 proteins, including PPP2R1A, was identified with a score of 6.6. Within this module, fourteen proteins were marked as hub genes: INTS1, UBE2S, FANCE, INTS11, PSMC3, HSPBP1, PSMD8, PNKP, FZR1, KDM5C, INTS5, RELA, SEC61A1, and TUFM (Fig. 2h). Venn diagram analysis revealed ten common proteins between the two PPI networks: PPP2R1A, POLR2C, POLR2D, INTS1, INTS5, INTS11, RELA, AXIN1, DVL2, and DCTN1 (Fig. 2i).

### Silencing *PPP2R1A* significantly reduces the proliferation of GC cell lines, whereas overexpressing *PPP2R1A* has little effect

**Fig. 3:**
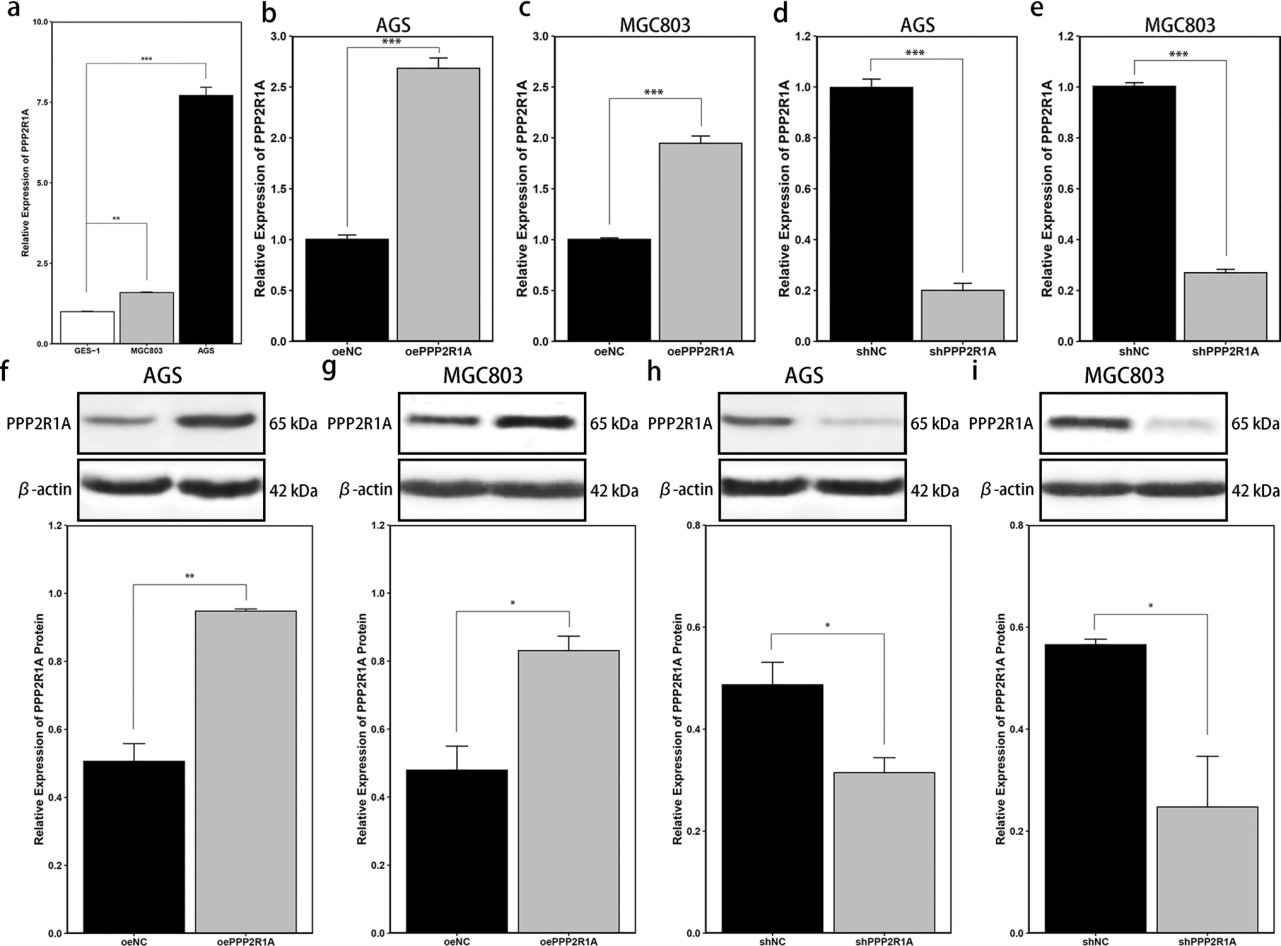
Validation of *PPP2R1A* knockdown and overexpression in GC cell lines. **a** mRNA levels of *PPP2R1A* in GES-1, AGS and MGC803 cells. **b**, **c** mRNA levels of *PPP2R1A* following its overexpression in AGS and MGC803 cells. **d**, **e** mRNA levels of *PPP2R1A* following its knockdown in AGS and MGC803 cells. **f**, **g** Protein levels of PPP2R1A following its overexpression in AGS and MGC803 cells. **h**, **i** Protein levels of PPP2R1A following its knockdown in AGS and MGC803 cells. The data are presented as the means ± standard deviations (SDs). Asterisks indicate statistically significant differences (*P < 0.05; **P < 0.01; ***P < 0.001). oeNC: Control group generated via the empty pLEX vector for overexpression experiments. shNC: Control group generated via the empty pLVX vector for shRNA experiments

RT-qPCR analysis of *PPP2R1A* mRNA expression in the AGS, MGC803, and GES-1 cell lines revealed significantly higher levels in AGS (*P* < 0.001) and MGC803 (*P* < 0.01) cells than in GES-1 cells (Fig. 3a).

To investigate the role of *PPP2R1A* in AGS and MGC803 cell lines, we constructed *PPP2R1A*-overexpressing and *PPP2R1A*-silenced cell lines. We then performed RT-qPCR to verify the efficiency of *PPP2R1A* overexpression (*P* < 0.001) (Fig. 3b, c) and silencing (*P* < 0.001) (Fig. 3d, e). Additionally, we conducted Western blot analysis to further confirm the overexpression (*P* < 0.05) (Fig. 3f, g) and silencing (*P* < 0.05) (Fig. 3h, i) of PPP2R1A.

**Fig. 4:**
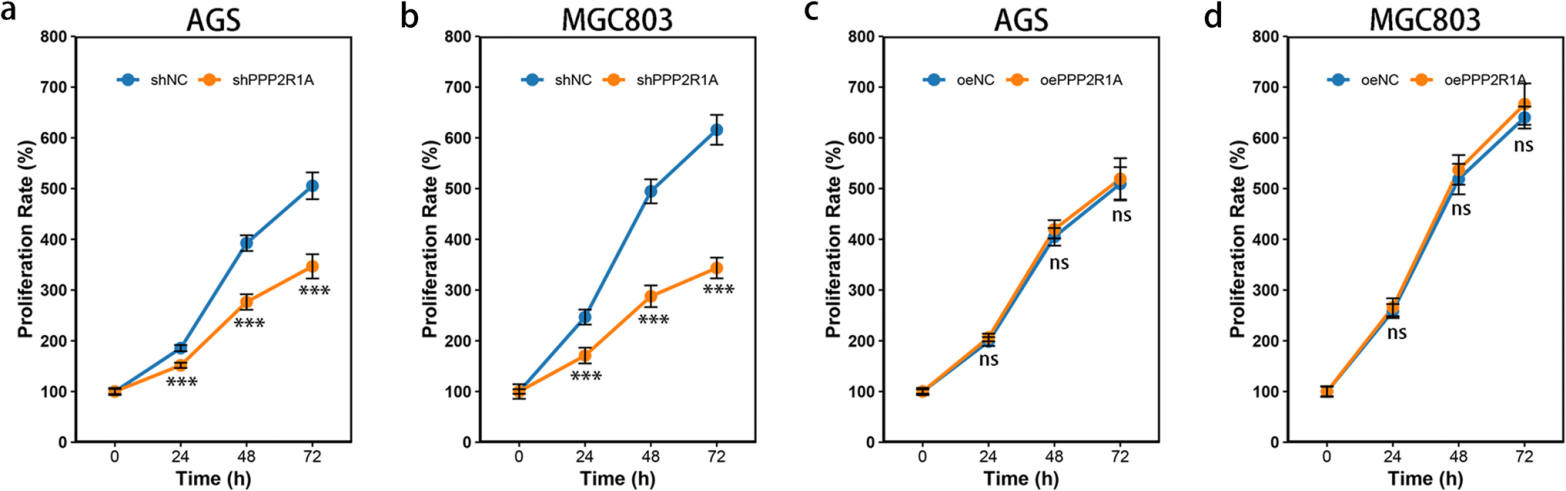
Effects of *PPP2R1A* expression on the proliferation of GC cell lines. **a**, **b** Proliferative capacity of AGS and MGC803 cells with *PPP2R1A* knockdown. **c**, **d** Proliferative capacity of AGS and MGC803 cells overexpressing *PPP2R1A*. The data are presented as the means ± SDs. Asterisks indicate statistically significant differences (*P < 0.05; **P < 0.01; ***P < 0.001)

To assess the proliferative capacity of the transfected GC cell lines, we employed a CCK-8 assay. The results indicated that the inhibition of *PPP2R1A* expression significantly reduced the proliferation of both the AGS and MGC803 cell lines (*P* < 0.001) (Fig. 4a, b). In contrast, the overexpression of *PPP2R1A* did not significantly affect the proliferation of these cell lines (*P* > 0.05) (Fig. 4c, d).

### Silencing *PPP2R1A* significantly reduces the migration and invasion of GC cell lines, whereas overexpressing *PPP2R1A* has little effect

**Fig. 5:**
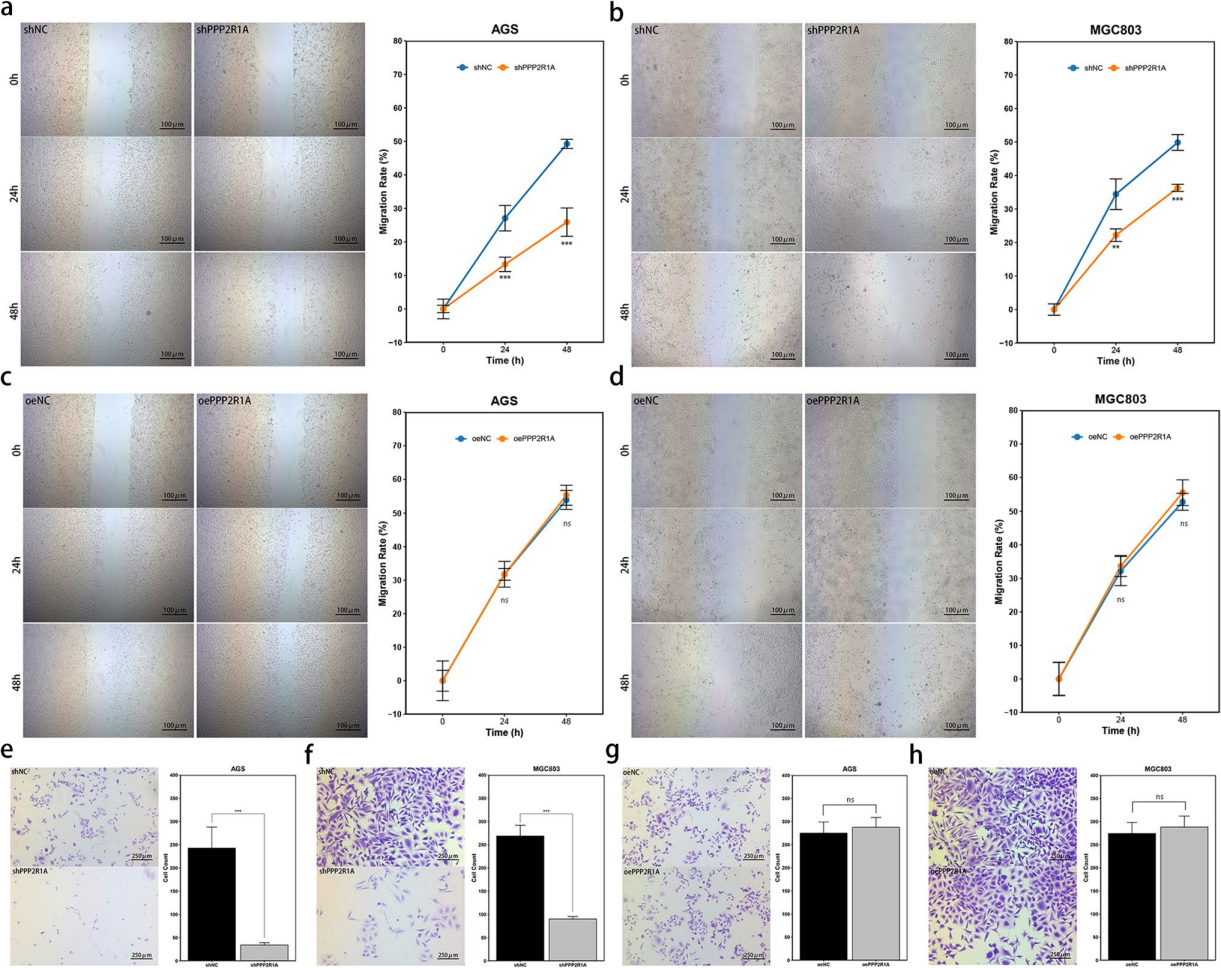
Effects of *PPP2R1A* expression on the migration and invasion capabilities of GC cell lines. **a**, **b** Migration abilities of AGS and MGC803 cells with *PPP2R1A* knockdown. Representative images are shown at 0, 24, and 48 h. **c**, **d** Migration abilities of AGS and MGC803 cells overexpressing *PPP2R1A*. Representative images are shown at 0, 24, and 48 h. **e**, **f** Migration and invasion capabilities of AGS and MGC803 cells with *PPP2R1A* knockdown. Representative images are displayed at 48 h. **g**, **h** Migration and invasion capabilities of AGS and MGC803 cells overexpressing *PPP2R1A*. Representative images are displayed at 48 h. The data are presented as the means ± SDs. Asterisks indicate statistically significant differences (*P < 0.05; **P < 0.01; ***P < 0.001)

To evaluate the migratory potential of the transfected GC cell lines, a scratch assay was performed. The results demonstrated that silencing *PPP2R1A* significantly inhibited the migration of both AGS and MGC803 cells (*P* < 0.01) (Fig. 5a, b). In contrast, the overexpression of *PPP2R1A* had a minimal effect on the migration of these cell lines (*P* > 0.05) (Fig. 5c, d).

Additionally, a Transwell assay was performed to assess the migration and invasion abilities of the transfected GC cell lines. The findings revealed that downregulation of *PPP2R1A* markedly impaired both the migration and invasion of AGS and MGC803 cells (*P* < 0.001) (Fig. 5e, f). Conversely, the overexpression of *PPP2R1A* had little effect on the migration and invasion of these cell lines (*P* > 0.05) (Fig. 5g, h).

### Silencing *PPP2R1A* significantly increases the apoptosis of GC cell lines, whereas overexpressing *PPP2R1A* has little effect

**Fig. 6:**
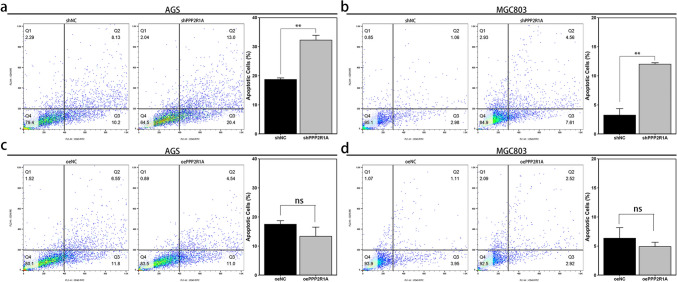
Effects of *PPP2R1A* expression on the apoptosis of GC cell lines. **a**, **b** Apoptotic capacity of AGS and MGC803 cells with *PPP2R1A* knockdown. Representative images of the quadrant plot are presented. **c**, **d** Apoptotic capacity of AGS and MGC803 cells overexpressing *PPP2R1A*. Representative images of the quadrant plot are presented. The data are presented as the means ± SDs. Asterisks indicate statistically significant differences (*P < 0.05; **P < 0.01; ***P < 0.001). Apoptotic cells: All Annexin V-FITC-positive cells (Q2 + Q3)

To assess the levels of apoptosis in the transfected GC cell lines, flow cytometry was conducted. The results demonstrated that silencing *PPP2R1A* significantly increased apoptosis in both the AGS and MGC803 cell lines (*P* < 0.01) (Fig. 6a, b). In contrast, the overexpression of *PPP2R1A* had a negligible effect on the apoptosis of these cell lines (*P* > 0.05) (Fig. 6c, d).

## Discussion

In this study, we conducted an in-depth investigation of the role of *PPP2R1A* in GC. The results indicated that *PPP2R1A* is significantly overexpressed in GC tissues, and that this overexpression is closely associated with poor patient survival. These findings suggest that *PPP2R1A* may play an important role in the occurrence and progression of GC.

*PPP2R1A*, the most prevalent scaffold subunit of the PP2A complex, has been implicated in the development, progression, and drug resistance of various malignancies through specific mutations (Verbinnen et al. 2021). The p.R183W mutation in *PPP2R1A* is associated with endometrial cancer development (Gonzalez-Bosquet et al. 2021) and confers increased resistance to clofarabine in uterine serous carcinoma cells through a gain-of-function mechanism (Remmerie et al. 2024). Additionally, this mutation enhances sensitivity to ATR inhibitors in ovarian clear cell carcinoma cells (Stewart et al. 2025). The P179R and S256F mutations drive the progression of high-grade uterine cancer (Haanen et al. 2025) and increase sensitivity to RNR inhibitors (O'Connor et al. 2022). Moreover, certain genetic variants in *PPP2R1A* are linked to an elevated risk of primary liver cancer (Wang et al. 2021). Given the regulatory role of *PPP2R1A* mutations in tumorigenesis, we analyzed the mutation status of *PPP2R1A* in STAD samples and found that there was no significant difference in expression levels between *PPP2R1A*-mutated and wild-type samples in GC. This suggests that upregulation of *PPP2R1A* expression, rather than mutations, is closely associated with the development of GC and poor prognosis in patients with GC.

Furthermore, our research demonstrated that silencing *PPP2R1A* significantly inhibited the proliferation, migration, and invasion of GC cells while promoting apoptosis. These findings indicate that *PPP2R1A* may play a role in promoting growth and metastasis in GC, potentially through its regulatory effects on cell cycle control (Hsin et al. 2021), apoptotic signaling pathways (Durmaz et al. 2025), and genes associated with cell migration (Wang et al. 2023). These findings further underscore the oncogenic properties of *PPP2R1A* in the progression of GC. However, this finding stands in contrast to the observations of Kang Q et al., who reported that a reduction in *PPP2R1A* expression facilitated the progression of B-ALL cells (Kang et al. 2024). Furthermore, the effects of *PPP2R1A* overexpression in GC cells differ from its role in promoting the proliferation of ovarian and endometrial cancer cells (Jeong et al. 2016), as does its ability to inhibit WNK1-induced cell migration in liver cancer cells (Hou et al. 2022). These results suggest that *PPP2R1A* may perform complex functions in cancer biology that are influenced by the cellular environment and its interactions with other molecules.

Surprisingly, the overexpression of *PPP2R1A* did not further promote the progression of GC cells, prompting a deeper investigation into the underlying mechanisms. Given that *PPP2R1A* is already highly expressed in GC, it is possible that a saturation state is reached when its expression reaches a certain threshold. Beyond this threshold, further upregulation of *PPP2R1A* may not significantly enhance the activity of PP2A. Additionally, PP2A activity is influenced by various endogenous and exogenous factors (Fujiki et al. 2023), and the simple upregulation of *PPP2R1A* may not be sufficient to overcome these inhibitory effects. Research has shown that the phosphorylation status of *PPP2R1A* can affect the interaction of *PPP2R1A* with PP2A and other regulatory factors, thereby modulating PP2A function (Zhou et al. 2025). Therefore, the overexpression of *PPP2R1A* may not significantly enhance PP2A activity in the specific biochemical context of GC cells. Moreover, considering the complex interplay among signaling pathways and the adaptability of cancer cells (Cordani et al. 2024), GC cell lines may have adapted to the high *PPP2R1A* environment by modulating other signaling pathways, thereby counteracting the effects of *PPP2R1A* upregulation on cell proliferation and survival. This adaptability may explain why the upregulation of *PPP2R1A* did not produce the expected significant effects.

Due to the current lack of foundational research on *PPP2R1A* in GC, we aimed to explore its potential molecular mechanisms by leveraging existing studies on PP2A. Extensive evidence indicated that PP2A activity is significantly reduced in GC, which is associated with the regulation of multiple cellular signaling pathways, such as the AKT/β-catenin pathway (Dai et al. 2021; Lin et al. 2024). The decrease in PP2A activity was primarily attributed to the overexpression of endogenous inhibitors of PP2A (Cai et al. 2023). Targeting these inhibitors was shown to be crucial for activating PP2A, inhibiting GC cell proliferation and migration, and inducing apoptosis (Wu et al. 2023; Liu et al. 2023). Additionally, the PP2A regulatory subunit STRN3 negatively regulates MST1/2, and targeting the PP2A-STRN3/4 complex to reactivate the Hippo pathway was significant for inhibiting GC and enhancing chemotherapy sensitivity (Cao et al. 2024; Tang et al. 2020). Moreover, silencing the PP2A regulatory subunit PPP2R2D reduces mTOR phosphorylation levels, thereby inhibiting GC cell proliferation and migration (Yu et al. 2018). These findings suggest that PP2A primarily regulates downstream signaling pathways in GC through dephosphorylation. Targeting its regulatory subunits is significant for inhibiting GC progression, implying that *PPP2R1A* may exert its potential oncogenic effects either by modulating PP2A activity or through PP2A-independent mechanisms.

Through functional network analysis of *PPP2R1A* co-expressed genes, we found that *PPP2R1A* is enriched in RNA splicing, and genes co-expressed with *PPP2R1A* are also enriched in the spliceosome pathway. Additionally, many key proteins interacting with PPP2R1A, such as POLR2E, INTS1, and SUPT5H, are closely related to transcriptional and post-transcriptional regulation. These findings suggest that the regulatory role of *PPP2R1A* may be associated with the splicing process. A 2018 report on alternative splicing analysis across tumors from 8,705 patients indicated that *PPP2R1A* may promote tumorigenesis and immune evasion by influencing splicing events (Kahles et al. 2018). Moreover, *PPP2R1A* also interacts with proteins involved in several signaling pathways, including AKT1 and AKT2, CDK4, RELA, and AXIN1 and DVL2. These interactions suggest that *PPP2R1A* may influence the biological functions of GC cells by participating in the regulation of these signaling pathways. However, the precise molecular regulatory mechanisms of *PPP2R1A* still require further experimental validation.

In summary, the overexpression of *PPP2R1A* is closely associated with poor survival rates in GC and significantly influence the proliferation, apoptosis, migration, and invasion of GC cells. This study has, for the first time, revealed the potential oncogenic characteristics of *PPP2R1A* in GC through bioinformatics analysis and in vitro cellular experiments. Given the limitations of our study to in vitro experiments and bioinformatics analysis, the specific molecular regulatory mechanisms of *PPP2R1A* have not been thoroughly explored. Future in-depth research employing gene-editing technologies, animal models, and clinical sample analyses will be essential to elucidate these mechanisms and to assess the potential of *PPP2R1A* as an independent prognostic biomarker or therapeutic target, thereby providing new directions for the clinical treatment of GC.

## Supplementary Information

Below is the link to the electronic supplementary material.

Supplementary file1 (TIF 123733 KB)

Supplementary file2 (TIF 132139 KB)

Supplementary file3 (TIF 127673 KB)

Supplementary file4 (TIF 123282 KB)

## Data Availability

The datasets used and analyzed during the current study are available from the corresponding author upon reasonable request.
